# État des lieux du traitement antirétroviral chez les personnes vivant avec le virus de l'immunodéficience humaine au Burkina Faso à l'ère de la stratégie « *test and treat* » de l'Organisation mondiale de la santé

**DOI:** 10.48327/mtsi.v5i2.2025.631

**Published:** 2025-06-25

**Authors:** Wedminère Noélie ZOUNGRANA-YAMEOGO, Christian YONLI, Toussaint COMPAORE, Fidèle BAKIONO, Arielle Rita BELEM, Luc DELMA, Abdoulaye SO, Ouo Mireille COULIBALY, Koiné Maxime DRABO

**Affiliations:** 1 Département de santé publique, Centre hospitalier universitaire de Tengandogo, Ouagadougou, Burkina Faso; 2 Secrétariat permanent du Conseil national de lutte contre le sida, Ouagadougou, Burkina Faso; 3 Service des maladies infectieuses, Centre hospitalier universitaire de Tengandogo, Ouagadougou, Burkina Faso; 4 Direction régionale de la région du Plateau-Central, Burkina Faso; 5 Service de la pharmacie hospitalière, Centre hospitalier universitaire de Tengandogo, Ouagadougou, Burkina Faso; 6 Direction de la qualité, Centre hospitalier universitaire de Tengandogo, Ouagadougou, Burkina Faso; 7 Institut de recherche en sciences de la santé, Ouagadougou, Burkina Faso

**Keywords:** VIH, PvVIH, Traitement antirétroviral, « *Test and treat* », Burkina Faso, Afrique subsaharienne, HIV, PLHIV, Antiretroviral treatment, Test and treat, Burkina Faso, Sub-Saharan AfricaIntroduction

## Abstract

**Introduction:**

La stratégie « *test and treat* » de l'OMS a contribué à augmenter de façon significative le nombre de personnes vivant avec le VIH (PvVIH) sous traitement antirétroviral (ARV). L'objectif de cette étude était de dresser un état des lieux du traitement ARV au Burkina Faso à l'ère de cette stratégie.

**Méthodes:**

Nous avons mené une étude rétrospective descriptive dans la région du Plateau-Central, l'une des 13 régions du pays. Les données annuelles de 2018 à 2023 ont été extraites de la base de dispensation pharmaceutique, base utilisée pour le suivi des personnes sous traitement ARV. Une tendance de mise sous traitement a été analysée. Les variables quantitatives ont été décrites en utilisant la médiane et l'intervalle interquartile et les variables qualitatives en utilisant la proportion.

**Résultats:**

De 2018 à 2023, la proportion annuelle des nouvelles personnes mises sous traitement par rapport à celles testées positives est passée de 25 % à 100 %. Chez les adultes, plus de 70 % étaient des femmes au cours de cette période. L’âge médian au début de traitement variait entre 35 ans [28-44] et 32 ans [25-44]. La durée médiane de traitement variait entre 5 ans [2-8] et 6 ans [3-12]. La proportion d'adultes ayant un niveau de dispensation en ARV d'au moins 95 % avait évolué en dents de scie avec un maximum à 70 % (2020) et un minimum à 47 % (2023). La principale combinaison thérapeutique utilisée chez les adultes était TDF/FTC/EFV 42 % en 2018, 50 % en 2019 et 38 % en 2020. La combinaison TDF/3TC/EFV était dominant en 2021, 46 %. En 2022 et 2023 la combinaison TDF/3TC/DTG était majoritaire, 76 % en 2022 et 91 % en 2023. Chez les enfants, le sexe masculin était dominant (autour de 55 %) de 2019 à 2022. L’âge médian au début du traitement variait entre 2 ans [0-9] et 4 ans [2-9], la durée médiane de traitement entre 5 ans [2-8] et 6 ans [3-12]. L'AZT/3TC/NVP était prédominant de 2018 à 2021 : 57 %, 59 %, 40 %, 40 %, et l'ABC/3TC/DTG à partir de 2022 : 52 % et 84 %. De 2018 à 2023, la proportion d'enfants ayant un niveau de dispensation en ARV d'au moins 95 % a évolué en dents de scie avec un maximum à 76 % (2019) et un minimum à 24 % (2023).

**Conclusion:**

La proportion des personnes mises sous traitement ARV a progressivement augmenté depuis les recommandations de « *test and treat* » de l'OMS. Ces résultats rapprochent le Burkina Faso de l'atteinte des objectifs de l'ONUSIDA.

## Abstract (en)

**Introduction:**

The World Health Organization's (WHO) “test and treat” strategy has significantly increased the number of people living with HIV (PLHIV) who receive antiretroviral therapy (ART). This study aimed to evaluate the status of ART in Burkina Faso during this period.

**Methods:**

A retrospective descriptive study was conducted in Plateau-Central, one of the country's 13 regions. We extracted annual data from 2018 to 2023 from the pharmaceutical dispensing database, which is used to monitor individuals receiving ART. We analyzed a trend in treatment initiation. Quantitative variables were described using the median and interquartile range, and qualitative variables were described using proportions.

**Results:**

From 2018 to 2023, the proportion of people who started treatment each year compared to the number of people who tested positive increased from 25% to 100%. Among adults during this period, more than 70% were women. The median age at the start of treatment ranged from 35 years (28–44) to 32 years (25–44). The median treatment duration ranged from five years [2-8] to six years [3-12]. The proportion of adults with at least 95% ART dispensing coverage fluctuated, ranging from a maximum of 70% in 2020 to a minimum of 47% in 2023. The main treatment combination used in adults was TDF/FTC/EFV, accounting for 42% in 2018, 50% in 2019, and 38% in 2020. The TDF/3TC/EFV combination was dominant in 2021, accounting for 46%. In 2022 and 2023, the TDF/3TC/DTG combination was the most common, at 76% in 2022 and 91% in 2023. Among children, males were predominant (around 55%) from 2019 to 2022. The median age at treatmentinitiation ranged from two years [0-9] to four years [2-9], and the median treatment duration ranged from five years [2-8] to six years [3-12]. AZT/3TC/NVP was predominant from 2018 to 2021 (57%, 59%, 40%, and 40%), and ABC/3TC/DTG was predominant from 2022 (52% and 84%). From 2018 to 2023, the proportion of children with at least 95% ART dispensing coverage fluctuated, ranging from a maximum of 76% in 2019 to a minimum of 24% in 2023.

**Conclusion:**

The proportion of people on ART has gradually increased since the WHO's “test and treat” recommendations. These results bring Burkina Faso closer to achieving the UNAIDS targets.

## Introduction

L'ONUSIDA a dénombré 97 000 personnes vivant avec le VIH (PvVIH) au Burkina Faso en 2023, dont 56 000 femmes et 10 000 enfants. « La transmission de la mère à l'enfant reste une préoccupation majeure » [16]. L'accessibilité aux antirétroviraux (ARV) a longtemps constitué un défi, en particulier dans les pays à faible revenu, en raison de leur coût élevé. Seules les personnes issues de classes sociales aisées pouvaient en bénéficier et leur mise sous traitement nécessitait des conditions immunitaires strictes. L'ONUSIDA a fixé un objectif ambitieux dans sa stratégie « 95-95-95 », dont le deuxième 95 vise à ce que 95 % des personnes connaissant leur statut sérologique suivent un traitement antirétroviral vital [15]. Toutefois, des efforts mondiaux et nationaux ont progressivement permis d'améliorer l'accès aux ARV. La gratuité du traitement par ARV au Burkina Faso a été adoptée par arrêté en 2010 [18]. Depuis 2016, l'OMS, à travers sa stratégie « *test and treat* », recommande de traiter toute personne dépistée positive au VIH, indépendamment du stade clinique ou du taux de CD4 [13]. Cette approche a entraîné une augmentation significative du nombre de PvVIH sous traitement ARV. À la fin décembre 2023, 30,7 millions de PvVIH [27-31,9 millions] bénéficiaient d'une thérapie antirétrovirale, contre 7,7 millions [6,7-8 millions] en 2010 [10]. Selon l'annuaire statistique du ministère de la Santé du Burkina Faso, 80 739 PvVIH étaient suivies dans les files actives, dont 99,8 % sous traitement ARV (TARV) en 2023 [6] contre 60 224 dont 52,36 % (31 532/60 224) sous TARV en 2010 [18]. La séroprévalence du VIH/sida dans la population adulte a considérablement diminué, passant de 2 % en 2010 [7] à 0,60 % en 2023 [6] grâce à la mise à disposition des ARV [14]. Depuis la mise en œuvre de la stratégie « *test and treat* », aucune étude n'a évalué l’état des lieux des TARV au Burkina Faso. L'objectif général de notre étude était de combler cette lacune en explorant l’état actuel de ces traitements. Il s'est agi plus spécifiquement de déterminer la proportion de personnes dépistées positives mises sous traitement, de décrire les caractéristiques des personnes incluses dans les files actives et de présenter l’évolution des intentions de traitement (ligne de traitement) et des protocoles thérapeutiques utilisés.

## Matériels et méthodes

Le Burkina Faso est un pays situé en Afrique de l'Ouest. Sa population était estimée à 20 505 155 habitants selon le dernier recensement général de la population et de l'habitation [4]. Il est classé 184^e^ sur 191 pays à l'Indice de développement humain 2021–2022 du PNUD, et plus de 40 % des Burkinabè vivent en-dessous du seuil de pauvreté [16]. Le pays est divisé en treize régions. Notre étude a été menée dans l'une des treize régions du pays, la région du Plateau-Central, dont la population était estimée à 1 116 799 en 2023 [7]. Cette région a une proximité avec la région du Centre et dispose d'une infrastructure de soins incluant plusieurs centres de santé offrant des services de prise en charge pour les PvVIH. Par ailleurs, elle n'est pas confrontée au défi sécuritaire : toutes les formations sanitaires étant ouvertes, elle était donc facilement accessible. Elle compte quatre files actives réparties dans trois districts sanitaires (Boussé, Ziniaré et Zorgho) et au niveau du Centre hospitalier régional (CHR). Les inclusions au CHR ont débuté en 2022, mais notre étude s'est concentrée sur les trois districts. En effet, le CHR n'avait que 19 patients dont les données n’étaient pas correctement enregistrées sur le fichier de dispensation qui comportait beaucoup de données manquantes. Les personnes enrôlées proviennent des zones urbaines et rurales, et parfois des zones hors aires sanitaires. Les services de diagnostic et de dépistage volontaire du VIH ont été mis en place entre 2006 et 2007 dans la région du Plateau-Central. Les files actives sont des cohortes ouvertes avec des mouvements d'entrées et de sorties des personnes. Les sorties peuvent être des transferts d'une file active d'une région à une autre ou au sein même de la même région d'un district à un autre, à la demande de la personne. Elles peuvent aussi être des perdus de vue ou des décès, des abandons et des arrêts de traitement. Dans le contexte du « *test and treat*», selon les directives du ministère de la Santé, le traitement doit être initié chez toute personne dépistée séropositive au VIH au plus tard dans les sept jours suivant le dépistage et la confirmation du diagnostic. Dans certains cas particuliers tels que le début du traitement d'une tuberculose non méningée, l'infection opportuniste à *Pneumocystis jiroveci,* la toxoplasmose, l'infection à cytomégalovirus, des infections dues au virus du groupe herpès, il faut initier le traitement ARV dans les deux semaines suivant le début du traitement et attendre au moins quatre semaines en cas de tuberculose et cryptococcose neuroméningée. Le traitement ARV doit être commencé sans délai en cas de complication liée au VIH ou d'une infection ne relevant d'aucun traitement spécifique.

Une étude rétrospective transversale descriptive a été conduite. L’échantillonnage a été exhaustif, incluant tous les patients figurant dans les files actives des trois districts (Boussé, Ziniaré et Zorgho) du Plateau-Central, et dont les informations étaient enregistrées dans le fichier de dispensation de la pharmacie. La pharmacie joue un rôle central dans le suivi des patients vivant avec le VIH. Elle assure non seulement la dispensation des médicaments, mais utilise également un outil spécifique pour leur suivi.

Les données sur le nombre de patients dépistés proviennent des annuaires statistiques du ministère de la Santé (2018 à 2023), et sont listées par région. S'agissant du dépistage, certaines données des associations de lutte contre le VIH ne sont pas prises en compte car non transmises (problèmes d'organisation, manque de compétence ou de communication). Les données sur le traitement ont été extraites à partir du fichier de dispensation de la pharmacie pour la période allant du 1^er^ janvier 2018 au 31 décembre 2023.

Ce fichier contient des informations détaillées sur chaque patient : informations générales -identifiant, district, année, nom, prénom, numéro de dossier, sexe, date de naissance, adresse/provenance, date d'inclusion, district sanitaire, calendrier de dispensation-, informations cliniques -type de VIH, motifs de mise sous traitement, type de patient-, informations biologiques - charge virale-, données sur les traitements - date de démarrage, ligne de traitement, protocole ARV, chimioprophylaxie (cotrimoxazole, dapsone, Rifapentine Isoniazide pendant trois mois) -, mode de dispensation des médicaments, dispositifs d'aide à l'observance chez les adultes - ravitaillement à six mois (RAVI6M) et ravitaillement communautaire hors structure de soins (RACODESS) -, évolution du patient. Dans le fichier des enfants il n'existe pas d*'items* concernant le RAVI6M et le RACODESS.

Le ravitaillement RAVI6M tout comme le RACODESS sont des approches différenciées des services du VIH. Elles constituent des stratégies d'aide à l'observance dans les centres de prises en charge des PvVIH recommandées par l'OMS. Le Burkina Faso a mis ces approches en œuvre sous forme pilote en 2020 [19]. Après cette première expérience qui a concerné 31 sites de prise en charge, la mise à l'échelle a suivi avec la formation des agents des 90 autres sites de prise en charge des PvVIH du pays en décembre 2021. L'année 2022 a donc été l'année de la mise en œuvre pratique. Le RAVI6M s'applique plus particulièrement aux personnes âgées de plus de 12 ans et stables, c'est-à-dire les PvVIH ayant reçu un traitement pendant au moins un an, qui ne présentent aucune infection opportuniste et qui ont des signes de succès thérapeutique (charge virale inférieure à 1 000 copies/ml). La personne est incluse dans cette stratégie sur la base des résultats de sa dernière charge virale. À défaut, les critères cliniques et immunologiques peuvent être appliqués. La personne bénéficiant de cette stratégie se rend dans une formation sanitaire tous les six mois pour un réapprovisionnement de son traitement antirétroviral. Après un an de stabilité sous RAVI6M, la visite clinique et la charge virale sont réalisées une fois par an. À partir de ce moment, les patients peuvent se réapprovisionner après six mois dans un endroit de réapprovisionnement alternatif, soit dans la structure sanitaire, soit dans la communauté. Le RACODESS est plutôt centré sur la communauté et comprend trois modalités : les postes de distribution (PODI), les groupes de patients TARV (GPT) et les dispensations communautaires par agents médicaux et non médicaux (DICOM). Il s'applique également aux personnes stables. Ont été inclus dans notre étude toutes les personnes (adultes et enfants) disposant des informations générales et médicales complètes pour les années de suivi. Les variables analysées ont concerné les données démographiques (âge, sexe,) les données cliniques (type de VIH, type de patient, ligne de traitement, protocole de traitement, durée de traitement, suivi, charge virale) et les données sur la dispensation : calendrier de dispensation, proportion de dispensation, RAVI6M, RACODESS. Les données ont été vérifiées pour garantir leur qualité. Le contrôle de qualité consiste à rechercher et à corriger les incohérences, erreurs de saisie, données aberrantes et doublons.

Certains transferts au sein de la même région (d'un district à un autre) peuvent être à l'origine de doublons mais ces cas sont rares. En général, les transferts sont plus courants d'une région à une autre. À partir des codes attribués aux personnes lorsqu'il y a des transferts dans la même région, ces personnes sont repérées et les données sont harmonisées afin d'éviter les doublons.

Les données ont été analysées avec le logiciel Epi-Info 7.2.6. Les paramètres descriptifs ont été calculés : la médiane et l'intervalle interquartile pour les variables quantitatives, la proportion pour les variables qualitatives. Un nouvel *item* a été ajouté pour calculer la proportion de dispensation par individu, calculée en divisant le nombre de dispensations réalisées par individu par le nombre de dispensations prévues. Par exemple, pour un patient débutant son traitement en janvier, 12 dispensations sont attendues sur une année complète. Nous avons obtenu l'autorisation du secrétaire général du ministère de la Santé du Burkina Faso pour faire ce travail dans la région du Plateau-Central. Toutes les dispositions ont été prises pour garantir la confidentialité des informations figurant dans la base de données, le fichier de dispensation de la pharmacie de la région du Plateau-Central.

## Résultats

Le nombre de personne suivies dans les files actives de 2018 à 2023 était respectivement de 1 780, 1 668, 1 883, 1 999, 2 068 et 2 383 chez les adultes et de 76, 80, 86, 92, 104, 127 chez les enfants. Le nombre de personnes dépistées positives au VIH de 2018 à 2023 était respectivement chacune de ces années de 681, 255, 229, 283, 233 et 256. Quant aux personnes nouvellement mises sous traitement, leur nombre était de 2018 à 2023 respectivement de 168, 184, 204, 115, 177 et 296. Les proportions des nouvelles personnes (adultes et enfants) mises sous traitement par rapport à celles désistées positives (adultes et enfants) sont illustrées par la Figure 1. Chez les adultes, l'âge médian avec l'intervalle interquartile au début de traitement (chez les nouveaux cas) de 2018 à 2023 étaient respectivement de 35 ans [28-44], 35 [28-44], 34 [28-46], 35 [26-45], 33 [26-26], 32 [25-44]. L’évolution des autres caractéristiques des adultes est présentée dans le tableau I.

**Figure I:**
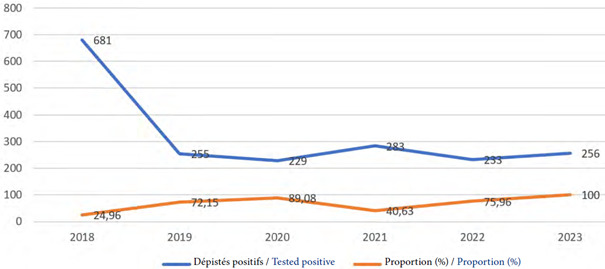
Évolution du nombre de personnes mises sous traitement par rapport au nombre de personnes dépistées positives au VIH de 2018 à 2023, région du Plateau-Central

**Tableau I:** Évolution des caractéristiques des adultes vivant avec le VIH de 2018 à 2023 dans la région du Plateau-Central, Burkina Faso

Année (Effectifs)	Âge médian file active (ans)	Sexe			Type VIH			
F n (%)	M n (%)	Manquant n (%)	VIH1 n (%)	VIH2 n (%)	VIH1 & 2 n (%)	Manquant n (%)
2018 (1 780)	40 [33-48]	1 322 (74)	458 (26)	0 (0)	1 635(92)	93 (5)	48 (3)	4 (0)
2019 (1 668)	41 [34-49]	1 239 (74)	408 (25)	21 (1)	1 503 (90)	83 (5)	46 (3)	36 (2)
2020 (1 883)	41 [33-50]	1 400 (74)	469 (25)	14 (1)	1 703 (91)	99 (5)	62(3)	19 (1)
2021 (1 999)	42 [34-50]	1 488 (75)	502 (25)	0 (0)	1 826 (91)	102 (5)	61 (3)	10 (1)
2022 (2 068)	43 [34-52]	1 542(75)	519 (25)	0 (0)	1 889 (91)	97 (5)	50(3)	25 (1)
2023 (2 383)	43 [34-52]	1 767(74)	586 (25)	0 (0)	2 190 (92)	100 (5)	37 (2)	26 (1)

F= féminin; M= masculin

L’évolution des mouvements des adultes est présentée dans le tableau II.

**Tableau II:** Évolution des mouvements des adultes dans les files actives de 2018 à 2023

Année (Effectifs)	Type de patients				Issue					
Nouveau n (%)	Ancien n (%)	Transfert entrant n (%)	Manquant n (%)	Vivant n (%)	Dédécé (%)	Perdus de vue (%)	Abandon n (%)	Transfert sortant n (%)	Arrêt du traitement n (%)
2018 (1 780)	168 (9)	1 067 (60)	19 (1)	526 (30)	1 727 (97)	21 (1)	24 (1)	0 (0)	8 (0)	0 (0)
2019 (1 668)	184 (11)	859 (52)	17 (1)	608 (36)	1 636 (98)	14 (1)	3 (0)	0 (0)	15 (1)	0 (0)
2020 (1 883)	204 (11)	1 647 (88)	15 (1)	18 (1)	1 867 (99)	6 (0)	3 (0)	0 (0)	7 (0)	0 (0)
2021 (1 999)	115 (6)	1 868 (94)	10 (1)	6 (0)	1 887 (94)	20 (1)	69 (3)	3 (0)	19 (1)	1 (0)
2022 (2 068)	177 (9)	1 842 (89)	26 (1)	223 (1)	2 010 (99)	17 (1)	23 (1)	0	18 (1)	0
2023 (2 383)	296 (12)	2019 (86)	27 (1)	18 (1)	2 255 (95)	37 (2)	52 (2)	1 (0)	38 (2)	0

Chez l'adulte, la proportion des personnes ayant au moins un taux de dispensation de 95 %, était, de 2018 à 2023, respectivement de 61,4 %, 63,6 %, 70,3 %, 65,9 %, 57,30 % et 46,8 %. La durée médiane de traitement en (années) et l'intervalle interquartile étaient sur cette même période respectivement de 5 [2-8], 5 [2-9], 5 [2-9], 6 [3-10],7 [3-12], 6 [3-12]. La première intention de traitement a été utilisée de 2018 à 2023 respectivement chez 1 701 personnes (95,6 %), 996 (60,2 %), 1 845 (98,7 %), 1 965 (98,8 %), 2023 (98,6 %), 2 324 (98,8 %). Les combinaisons thérapeutiques ont évolué sur les 5 ans. Le tableau III présente les détails de l’évolution des combinaisons thérapeutiques utilisées de 2018 à 2023. Les données sur le RAVI6M et le RACODESS n’étaient présentes dans la base qu'en 2023. Le RAVI6M a concerné 705 personnes, soit 29,5 % (705/2 383) et le RACODESS 231 patients, soit 10 % (231/2 383).

**Tableau III:** Évolution des combinaisons thérapeutiques utilisées chez les adultes de 2018-2023 dans la région du Plateau-Central, Burkina Faso, sur l'ensemble de la file active

Année	Protocole 1	Protocole 2	Protocole 3	Protocole 4
2018 (1 780)	TDF/FTC/EFV1 (41,9 %)	AZT/3TC/NVP3 (30,8 %)	AZT/3TC/EFV6 (9,7 %)	ABC/3TC/EFV8 (0,1 %)
2019 (1 668)	TDF/FTC/EFV (49,6 %)	AZT/3TC/NVP (25,3 %)	TDF/FTC/NVP (4,6 %)	AZT/3TC/EFV (4 %)
2020 (1 883)	TDF/FTC/EFV (38,2 %)	TDF/3TC/DTG (33,1 %)	TDF/3TC/EFV4 (17,4 %)	TDF/FTC/EFV (3,7 %)
2021 (1 999)	TDF/3TC/EFV (45,7 %)	TDF/3TC/DTG (44,7 %)	TDF/FTC/EFV (3,5 %)	TDF/FTC+LPV/r (2,3 %)
2022 (2 068)	TDF/3TC/DTG (76,2 %)	TDF/3TC/EFV (23,6 %)	ABC/3TC/DTG7 (0,2 %)	ABC/3TC/EFV (0,7 %)
2023 (2 383)	TDF/3TC/DTG (90,6 %)	TDF/3TC/EFV (8,9 %)	ABC/3TC/DTG (0,1 %)	ABC/3TC/EFV (0,1 %)

TDF/FTC/EFV : Traitement ARV combinant Tenofovir plus Emtricitabine plus Efavirenz

TDF/3TC/DTG : Traitement ARV combinant Tenofovir plus Lamivudine plus Dolutegravir

AZT/3TC/NVP : Traitement ARV combinant Zodovudine plus Lamivudine plus Nevirapine

TDF/3TC/EFV : Traitement ARV combinant Tenofovir plus Lamivudine plus Efavirenz

AZT/3TC/EFV : Traitement ARV combinant Zidovudine plus Lamivudine plus Efavirenz

ABC/3TC/DTG : Traitement ARV combinant Abacavir plus Lamivudine plus Dolutegravir

ABC/3TC/EFV : Traitement ARV combinant Abacavir plus Lamivudine plus Efavirenz

TDF/FTC+LPV/r : Traitement ARV Tenofovir plus Emtricitabine plus Lopinavir /Ritonavir

TDF/FTC/NVP : Traitement ARV combinant Tenofovir plus Emtricitabine plus Nevirapin

Le nombre d'enfants suivis dans les files actives de 2018 à 2023 était respectivement de 76, 80, 86, 92, 104 et 127. Les caractéristiques de ces enfants sont présentées dans le tableau IV.

**Tableau IV:** Évolution des caractéristiques des enfants vivant avec le VIH de 2018 à 2023

Année (Effectifs)	Âge médian file active (ans) [IQR]	Sexe			Type VIH			
F n (%)	M n (%)	Manquant n (%)	VIH1 n (%)	VIH2 n (%)	VIH1 & 2 n (%)	Manquant n (%)
2018 (76)	9 [5-11]	37 (49)	34 (45)	5 (1)	69 (95)	4 (6)	0 (0)	3
2019 (80)	9 [5-11]	34 (43)	41 (51)	5 (6)	73 (97)	2 (3)	0 (0)	5
2020 (86)	8 [5-11]	40(47)	45 (52)	1 (1)	82 (97)	3 (4)	0 (0)	1
2021 (92)	8 [4-11]	38(41)	52 (57)	2 (2)	88 (97)	3 (3)	0 (0)	1
2022 (104)	8 [4-12]	42(40)	53 (51)	9 (9)	100 (98)	2 (2)	0 (0)	2
2023 (127)	10 [5-14]	57 (45)	57 (45)	10 (8)	121 (99)	1 (1)	0 (0)	5

F= féminin; M= masculin; IQR : intervalle interquartile

Les mouvements des enfants dans la file active sont présentés dans le tableau V.

**Tableau V:** Évolution des mouvements des enfants dans les files actives de 2018 à 2023

Année (Effectifs)	Type de patients				Issue					
Nouveau n (%)	Ancien n (%)	Transfert entrant n (%)	Manquant n (%)	Vivant n (%)	Dédécé (%)	Perdus de vue (%)	Abandon n (%)	Transfert sortant n (%)	Arrêt du traitement n (%)
2018 (76)	8 (11)	64 (89)	0 (0)	4 (5)	75 (99)	1 (1)	0 (0)	0 (0)	0 (0)	0 (0)
2019 (80)	12 (16)	62 (82)	2 (3)	4 (5)	79 (99)	1 (1)	0 (0)	0 (0)	0 (0)	0 (0)
2020 (86)	14 (18)	65 (81)	1 (1)	6 (7)	85 (99)	1 (1)	0 (0)	0 (0)	0 (0)	0 (0)
2021 (92)	8 (9)	84 (92)	0 (0)	0 (0)	88 (96)	3 (3)	0 (0)	0 (0)	1 (1)	0 (0)
2022 (104)	22 (22)	78 (78)	0 (0)	4 (4)	97 (93)	4 (4)	1 (1)	0 (0)	2 (2)	0 (0)
2023 (127)	37 (30)	87 (70)	0 (0)	3(2)	121 (95)	3 (2)	2 (2)	0 (0)	1 (1)	0 (0)

Chez les enfants, la proportion des personnes ayant au moins un taux de dispensation de 95 %, de 2018 à 2023, était respectivement 41,1 %, 75,6 % %, 68,6 %, 66,3 %, 63,3 % et 24,2 %. La première intention de traitement a été utilisée de 2018 à 2023 respectivement chez 55 personnes (77 %), 64 (85 %), 74 (99 %), 79 (87 %), 86 (91 %), 85 (90 %). L’âge médian et l'intervalle interquartile en début du traitement de 2018 à 2023 étaient respectivement 2 [0-9], 4 [1-7], 2 [0-9], 5[2-10], 3 [2-7], 4 [2-9]. La durée médiane de traitement de 2018 à 2023 était respectivement 3 [2-6], 44 [4-6], 4 [2-6], 4 [2-6], 4 [2-7], 4 [1-8].

Les combinaisons thérapeutiques ont évolué de 2013 à 2023. Le tableau VI donne les détails sur l’évolution des combinaisons thérapeutiques utilisées chez les enfants de 2018 à 2023.

**Tableau VI:** Évolution des combinaisons thérapeutiques utilisées de 2018 à 2023 chez les enfants

Année	Protocole 1	Protocole 2	Protocole 3	Protocole 4
2018 (76)	AZT/3TC/NVP (56,7 %)	ABC/3TC/EFV (21,7 %)	ABC/3TC/LPV/r (8,3 %)	ABC/3TC/LPV/r (8,3 %)
2019 (80)	AZT/3TC/NVP (58,7 %)	ABC/3TC/EFV (17,3 %)	ABC/3TC/LPV/r (17,3 %)	ABC/3TC/NVP (4 %)
2020 (86)	AZT/3TC/NVP (40,3 %)	ABC/3TC/EFV (24,4 %)	ABC/3TC/LPV/r (19,5 %)	TDF/3TC/EFV (3,7 %)
2021 (92)	AZT/3TC/NVP (40,2 %)	ABC/3TC/LPV/r (24,1 %)	ABC/3TC/EFV (18,1 %)	TDF/3TC/DTG (6,9 %
2022 (104)	ABC/3TC/DTG (51,6 %)	AZT/3TC/NVP (18,3 %)	TDF/3TC/DTG (18,3 %)	ABC/3TC/LPV/r (9,6 %)
2023 (127)	ABC/3TC/DTG (84,3 %)	TDF/3TC/DTG (9 %)	ABC/3TC + LPV/r (2,3 %)	ABC /3TC/EFV (1,1 %)

TDF/FTC/EFV : Traitement ARV combinant Tenofovir plus Emtricitabine plus Efavirenz

TDF/3TC/DTG : Traitement ARV combinant Tenofovir plus Lamivudine plus Dolutegravir

AZT/3TC/NVP : Traitement ARV combinant Zodovudine plus Lamivudine plus Nevirapine

TDF/3TC/EFV : Traitement ARV combinant Tenofovir plus Lamivudine plus Efavirenz

ABC/3TC/DTG : Traitement ARV combinant Abacavir plus Lamivudine plus Dolutegravir

ABC/3TC/EFV : Traitement ARV combinant Abacavir plus Lamivudine plus Efavirenz

ABC/3TC/LPV/r : Traitement ARV combinant Abacavir plus Lamivudine plus Lopinavir / Ritonavir

ABC/3TC/NVP : Traitement ARV combinant Abacavir plus Lamivudine plus Nevirapine

## Discussion

Cette étude a permis de dresser le bilan du traitement antirétroviral chez les PvVIH (adultes et enfants) à l’ère de la stratégie « *test and treat*» de l'OMS au Burkina Faso, en prenant le cas de la région du Plateau-Central. Cette région n'est pas affectée par les défis sécuritaires auxquels le Burkina Faso est confronté depuis environ dix ans. Il n'y a pas eu de répercussions sur les activités de prise en charge des PvVIH ni des approvisionnements en intrants. Nous avons pu évaluer la proportion des personnes mises sous traitement, avoir un aperçu des différentes combinaisons thérapeutiques utilisées et voir le niveau de dispensation des médicaments au niveau de la pharmacie.

Cette étude présente des limites méthodologiques. Les données utilisées reposent sur les enregistrements des fichiers de dispensation, ce qui peut comporter des biais liés à des erreurs de saisie comme la date de naissance, la date de mise sous ARV. D'autre part, étant donné que nous n'avons pas travaillé avec les sources primaires de collecte de données, d'autres erreurs restent probables. Malgré l'existence d'une variable en lien avec la charge virale, les données la concernant sont inexistantes. Par ailleurs, des grèves dans le secteur ont beaucoup impacté les données en 2019.

L'objectif du deuxième 95 de l'ONUSIDA est que 95 % des personnes diagnostiquées avec le VIH soient mises sous traitement antirétroviral (TARV). Le « *test and treat*» a contribué à améliorer les résultats liés à cet objectif. Les résultats montrent une augmentation progressive de la proportion de personnes sous TARV de 25,84 % en 2018 à plus de 100 % en 2023. En effet, en 2023, le nombre de personnes mises sous traitement dépasse le nombre de personnes dépistées. Cela s'explique par le fait que certaines données sur le dépistage ne sont pas toujours transmises au niveau de la région pour compilation. Par ailleurs, certaines personnes dépistées hors de la région du Plateau-Central, pour des raisons de confidentialité ou de proximité, viennent s'enrôler dans la région pour le traitement. La mise en œuvre du « *test and treat »»* a rencontré des résistances initiales, surtout de la part de certains médecins qui n’étaient pas favorables à ce que les infirmiers initient le traitement ARV dans les sites de prise en charge sans un minimum de bilan biologique, notamment la créatininémie pour évaluer les risques de complications rénales. Par ailleurs, des résistances ont été souvent notées de la part des PvVIH elles-mêmes en raison de la crainte de stigmatisation et des évènements indésirables induits par les ARV souvent rapportés par d'autres PvVIH.

La proportion de patients sous traitement semble incohérente en 2021 (40,63 %). Cette situation serait liée à une insuffisance de captation provisoire des données dans le district sanitaire de Zorgho. L’âge médian à l'initiation du traitement variait entre 32 et 35 ans chez les adultes et 2 à 5 ans chez les enfants. Cette tranche d’âge chez les adultes est également rapportée par Essomba *et al.* dans une étude réalisée en 2012 au Cameroun sur 1 661 PvVIH [3]. L’âge d'enrôlement des enfants, légèrement en dessous de celui rapporté par Ngwej *et al.* dans une étude réalisée sur une cohorte de 72 enfants en 2015 au Congo [8] est tardif si toutefois le mode de contamination était vertical. Le dépistage des enfants n'est pas courant dans notre contexte. Leur statut sérologique est souvent découvert à l'occasion d'une infection opportuniste. Des actions de sensibilisation sur le dépistage précoce devraient être promues afin d'offrir une prise en charge optimale aux enfants. Par ailleurs, au vu des résultats positifs des programmes de prévention de la transmission mère-enfant du VIH à travers l'utilisation des thérapies antirétrovirales hautement actives, les femmes enceintes devraient être davantage encouragées à participer à ces programmes afin de réduire voire d’éliminer la transmission mère-enfant du VIH. Chez les adultes, le sexe féminin est majoritaire sur toute la période considérée. Cette tendance évolue en dents de scie chez les enfants. Cette prédominance féminine suit la tendance nationale où la prévalence du VIH est plus élevée chez les femmes que chez les hommes [9]. Elle a été également rapportée dans d'autres pays africains : Guinée, République démocratique du Congo, Cameroun [1, 3, 24]. Par ailleurs, il a été noté que les femmes vivant avec le VIH étaient davantage susceptibles d'avoir accès au dépistage et au traitement du VIH que les hommes [11, 12].

Des perdus de vue ont été enregistrés au cours des 5 années de l’étude chez les adultes avec une tendance à l'augmentation. Chez les enfants, les perdus de vue sont peu nombreux. Dans le cadre du suivi des PvVIH sous ARV, les perdus de vue ont toujours constitué un défi au Burkina Faso [2, 22, 23]. Les ARV sont des médicaments à prise quotidienne et à vie, avec des visites de suivi et des ravitaillements qui peuvent nécessiter plusieurs déplacements dans les formations sanitaires. Ce concept de file active peut être contraignant pour certains PvVIH qui, par la suite, quittent les files actives sans donner de nouvelles.

La létalité liée au VIH varie entre 1 à 2 % chez les adultes et 1 à 4 % chez les enfants. Les chiffres chez les adultes sont similaires aux données nationales et mondiales [5, 12]. Cependant, chez les enfants, ces chiffres sont plus élevés. Cette légère hausse pourrait éventuellement s'expliquer par le fait que certains enfants de notre étude commencent leur traitement ARV assez tard, surtout si leur contamination n'est pas verticale. Même si des décès sont toujours enregistrés chez les PvVIH, ceux-ci sont en baisse significative comparés aux létalités décrites dans certaines études il y a une dizaine d'années [17, 21, 22], témoignant de l'efficacité des TARV et de la qualité du suivi médical des patients. Ce faible taux illustre la sécurité des médicaments ARV et leur rôle crucial dans la réduction significative de la mortalité associée au VIH, confirmant leur impact dans les stratégies de lutte contre cette maladie. Le taux de dispensation d'au moins 95 % des TARV reste relativement modeste chez les adultes : audessus de 50 % entre 2019 et 2022 et autour de 50 % en 2022 et 2023. Cette tendance est similaire chez les enfants, ces derniers ayant atteint des taux de dispensation supérieurs à 60 % entre 2019 et 2022 et inférieurs à 50 % en 2023. Ces résultats indiquent une difficulté à maintenir une observance optimale dans la durée. Même si de nouvelles stratégies d'aide à l'observance telles que le RAVI6M et le RACODESS ont été mises en place à partir de 2022 pour améliorer l'observance des PvVIH, des efforts doivent être faits pour améliorer les taux de dispensation. Par ailleurs, des évaluations régulières de l'impact de ces stratégies sont nécessaires afin de mieux les adapter aux besoins des PvVIH.

Sur une période de six ans, notre étude a montré que la première intention de traitement a été utilisée majoritairement chez les adultes et chez les enfants, ce qui traduit une bonne tolérance aux ARV ne nécessitant pas de passage en deuxième ou troisième intention [14].

Les recommandations de l'OMS de 2013 indiquent pour le TARV de première intention d'associer deux inhibiteurs nucléosidiques de la transcriptase inverse à un inhibiteur non nucléosidique de la transcriptase inverse chez les patients infectés par le VIH-1 [20]. Les résultats de notre étude s'alignent parfaitement avec ces recommandations, confirmant leur pertinence dans notre contexte.

L’évolution des protocoles thérapeutiques entre 2018 et 2023 reflète une transition vers des molécules plus efficaces et mieux tolérées suivant les recommandations internationales [23]. Chez les adultes, une transition marquée vers le TDF/3TC/DTG est observée dès 2020, atteignant 90,55 % en 2023.

Chez les enfants, l'ABC/3TC + DTG devient dominant en 2023 (84,27 %). Cette évolution positive indique une bonne adoption des nouvelles directives. Cependant, une analyse continue des profils de résistance et des données d'observance pourrait guider l'optimisation des protocoles.

## Conclusion

Cette étude met en lumière des avancées significatives dans la lutte contre le VIH/sida, mais elle révèle également des défis majeurs en termes de mise en œuvre des directives, d’équité dans l'accès aux soins, et de maintien d'une observance optimale. Une attention particulière devra être accordée aux populations pédiatriques et aux stratégies d'observance pour garantir un impact durable dans la région.

## Remerciements

Nous remercions le Dr Issa Ouedraogo, secrétaire général du ministère de la Santé, pour avoir accordé l'autorisation de collecte des données, ainsi que les médecins chefs de district, les pharmaciens et les préparateurs d’État en pharmacie pour leur collaboration.

## Source de financement

Ce travail n'a bénéficié d'aucune source de financement.

## Contributions des auteurs et autrices

Le Dr Zoungrana-Yameogo est le concepteur de cette étude, qui a analysé, interprété les données et rédigé la première version, relue par Dr Christian Yonli. Ce texte a été partagé aux Drs Arielle Rita Belem, Fidèle Bakiono, Luc Delma, Mireille Ouo Coulibaly et Abdoulaye So pour lecture. Toussaint Compaoré a participé au traitement et à l'apurement de la base de données. Ce travail de recherche a été supervisé par le Pr Maxime Koiné Drabo qui a autorisé la publication du manuscrit.

## Conflits d'intérêts

Les auteurs ne déclarent aucun conflit d'intérêts.
