# Supplementary material for: Current status of antiretroviral treatment for people living with human immunodeficiency virus in Burkina Faso in the era of the World Health Organization's “test and treat” strategy
Source: Med Trop Sante Int. 2025 Jun 25;5(2):mtsi.v5i2.2025.631. [Article in French] doi: 10.48327/mtsi.v5i2.2025.631 (PMC12305227; doi:10.48327/mtsi.v5i2.2025.631)
Supplement: Related texts

# Research article: Current status of antiretroviral treatment for people living with human immunodeficiency virus in Burkina Faso in the era of the World Health Organization's “test and treat” strategy

## Introduction

According to UNAIDS, there were an estimated 97,000 people living with HIV (PLHIV) in Burkina Faso in 2023, including 56,000 women and 10,000 children. “Mother-to-child transmission remains a major concern” [16]. Access to antiretroviral drugs (ARTs) has long been challenging, especially in low-income countries, due to their high cost. Only people from wealthy social classes could benefit from them, and strict immune conditions were required to start treatment. In its “95-95-95” strategy, UNAIDS has set an ambitious target in its “95-95-95” strategy, the second 95 of which aims to ensure that 95% of people who know their HIV status are receiving life-saving ART [15]. However, global and national efforts have gradually improved access to ARTs. In 2010, free ART was adopted by decree in Burkina Faso [18]. Since 2016, the WHO has recommended treating anyone who tests positive for HIV, regardless of clinical stage or CD4 count, through its “test and treat” strategy [13]. This approach has led to a significant increase in the number of PLHIV on ART. As of December 2023, an estimated 30.7 million people were receiving antiretroviral therapy, up from 7.7 million in 2010 [10]. According to the Ministry of Health's 2023 statistical yearbook, 80,739 PLHIV were monitored in the active files, 99.8% of whom received ART [6], compared with 60,224 in 2010, 52.36% (31,532) of whom received ART [18]. Thanks to the availability of ART [14], the HIV/AIDS seroprevalence rate in the adult population has fallen significantly, from 2% in 2010 [7] to 0.60% in 2023 [6].

No study has assessed the status of ART in Burkina Faso since the implementation of the “test and treat” strategy. Our study aimed to address this gap by examining the current status of these treatments. Specifically, we aimed to determine the proportion of individuals who tested positive and received treatment, describe the characteristics of those included in the active files, and present the evolution of treatment intentions and therapeutic protocols.

## Materials and methods

Burkina Faso is a country located in West Africa. According to the latest general population and housing census, its population was estimated at 20,505,155 [4]. Burkina Faso is ranked 184^th^ out of 191 countries on the 2021–2022 Human Development Index by the UNDP, and over 40% of its population lives below the poverty line [16]. The country is divided into thirteen regions. Our study was conducted in the Plateau-Central region, one of the country's thirteen regions, which had an estimated population of 1,116,799 in 2023 [7]. The Plateau-Central region is close to the Central region and has a healthcare infrastructure that includes several health centers offering care services for people living with HIV/AIDS (PLHIV). Furthermore, the region does not face any security challenges, and all health facilities are open and easily accessible. There are four active files spread across three health districts (Boussé, Ziniaré, and Zorgho) and the Regional Hospital Center (RHC). Inclusions at the RHC began in 2022; however, our study focused on the three districts. This is because the RHC only had 19 patients, and much of the data in their dispensing files was missing or incorrectly recorded. Those enrolled come from urban and rural areas and sometimes from areas outside the health zones. Voluntary HIV diagnosis and screening services were established in the Plateau-Central region between 2006 and 2007. Active files are open cohorts with people entering and leaving. Discharges may involve transfers from one active file to another, either between regions or within the same region from one district to another, at the patient's request. Exits may also be due to loss to follow-up, death, dropout, or treatment discontinuation. According to Ministry of Health guidelines in the context of “test and treat,” treatment must be initiated in all individuals who test HIVpositive no later than seven days after screening and confirmation of diagnosis. In certain cases, such as when initiating treatment for non-meningitic tuberculosis, an opportunistic infection caused by *Pneumocystis jiroveci,* toxoplasmosis, cytomegalovirus infection, or an infection caused by a herpes virus, ART should be started within two weeks of beginning treatment. However, it should be delayed by at least four weeks in cases of tuberculosis and cryptococcal meningitis. ART should be started immediately for HIV-related complications or infections that do not require specific treatment.

A descriptive, cross-sectional, retrospective study was conducted. The sample included all patients on the active lists of the three districts (Boussé, Ziniaré, and Zorgho) of the Central Plateau, and their information was recorded in the pharmacy dispensing file. The pharmacy plays a central role in following up with patients living with HIV. They not only dispense medication, but also use a specific tool for patient follow-up.

Data on the number of patients screened comes from the Ministry of Health's statistical yearbooks from 2018 to 2023 and is listed by region. Regarding testing, some data from HIV associations were not considered because they were not transmitted due to organizational problems, a lack of expertise, or communication issues. Treatment data were extracted from the pharmacy dispensing file for the period from January 1, 2018, to December 31, 2023.

This file contains detailed information on each patient: General information: ID, district, year, surname, first name, file number, sex, date of birth, address/origin, date of inclusion, health district, dispensing schedule; clinical information: type of HIV, reasons for starting treatment, type of patient; biological information: viral load; treatment data: start date, treatment line, ART protocol, and chemoprophylaxis (cotrimoxazole, dapsone, rifapentine, and isoniazid for three months). The file also contains information on the method of drug dispensing and adherence support mechanisms for adults, such as six-month refills (RAVI6M) and community refills outside healthcare facilities (RACODESS). The children's file does not contain any items concerning RAVI6M or RACODESS.

RAVI6M refills and RACODESS are two different approaches to HIV services. They are adherence support strategies recommended by the WHO for use in PLHIV care centers. Burkina Faso implemented these approaches on a pilot basis in 2020 [19]. Following this initial experience involving 31 care sites, the program was scaled up in December 2021 with the training of an additional 90 PLHIV care sites across the country. Thus, 2022 was the year of practical implementation. RAVI6M is specifically designed for people over the age of 12 who are stable. This includes PLHIV who have been on treatment for at least one year, have no opportunistic infections, and show signs of treatment success (viral load below 1,000 copies/ml). A person is included in this strategy based on their most recent viral load results. If these results are unavailable, clinical and immunological criteria may be applied. Those benefiting from this strategy visit a health facility every six months to replenish their antiretroviral treatment. After one year of stability on RAVI6M, clinical visits and viral load testing are performed annually. After that, patients can obtain refills every six months from an alternative location, either a health facility or a community site. RACODESS is more community-based and comprises three modalities: point-of-distribution (POD) sites, ART patient groups (APGs), and community dispensing by medical and non-medical personnel (CDM). It also applies to stable individuals. Our study included all individuals (adults and children) with complete general and medical information for the follow-up period. The analyzed variables included demographic data (age and sex), clinical data (HIV type, patient type, treatment line, treatment protocol, treatment duration, follow-up and viral load), and dispensing data (dispensing schedule, dispensing proportion, and RAVI6M and RACODESS). The data were verified to ensure quality. Quality control involves identifying and correcting inconsistencies, data entry errors, outliers, and duplicates. While some transfers within the same region (from one district to another) may result in duplicates, these cases are rare. In general, transfers are more common between regions. When there are transfers within the same region, the codes assigned to individuals are used to identify them and harmonize the data to avoid duplicates.

The data were analyzed using Epi Info 7.2.6 software. Descriptive parameters were calculated, including the median and interquartile range for quantitative variables and the proportion for qualitative variables. A new item was added to calculate the proportion of dispensing per individual. This is calculated by dividing the number of dispenses per individual by the total number of planned dispenses. For instance, a patient starting treatment in January is expected to receive 12 doses over the course of a full year. We obtained authorization from the Secretary General of the Ministry of Health of Burkina Faso to conduct this study in the Plateau-Central region. All measures have been taken to ensure the confidentiality of the information contained in the Plateau-Central region pharmacy's dispensing file.

## Results

From 2018 to 2023, the number of people monitored in the active files was 1,780, 1,668, 1,883, 1,999, 2,068, and 2,383 among adults, and 76, 80, 86, 92, 104, and 127 among children. The number of people who tested positive for HIV from 2018 to 2023 was 681, 255, 229, 283, 233, and 256 for each year, respectively. The number of people who started treatment for the first time between 2018 and 2023 was 168, 184, 204, 115, 177, and 296, respectively. Figure 1 shows the proportions of new people (adults and children) who started treatment compared to those who discontinued treatment (adults and children).

**Figure I:** Change in the number of people receiving treatment compared to the number of people testing positive for HIV from 2018 to 2023, Plateau-Central region

In adults, the median age with interquartile range at the start of treatment (in new cases) from 2018 to 2023 was 35 years [28-44], 35 [28-44], 34 [28-46], 35 [26-45], 33 [26-26], 32 [25-44]. Changes in other characteristics of adults are shown in Table I. Changes in adult movements are presented in Table II.

**Table I:** Changes in the characteristics of adults living with HIV from 2018 to 2023 in the Plateau-Central region, Burkina Faso

Year (Headcount)	Median age of active patient file (years)	Gender			HIV type			
F n (%)	M n (%)	Missing data n (%)	HIV1 n (%)	HIV2 n (%)	HIV1 & 2	Missing data n (%)
2018 (1 780)	40 [33-48]	1 322 (74)	458 (26)	0 (0)	1 635(92)	93 (5)	48 (3)	4 (0)
2019 (1 668)	41 [34-49]	1 239 (74)	408 (25)	21 (1)	1 503 (90)	83 (5)	46 (3)	36 (2)
2020 (1 883)	41 [33-50]	1 400 (74)	469 (25)	14 (1)	1 703 (91)	99 (5)	62(3)	19 (1)
2021 (1 999)	42 [34-50]	1 488 (75)	502 (25)	0 (0)	1 826 (91)	102 (5)	61 (3)	10 (1)
2022 (2 068)	43 [34-52]	1 542(75)	519 (25)	0 (0)	1 889 (91)	97 (5)	50(3)	25 (1)
2023 (2 383)	43 [34-52]	1 767(74)	586 (25)	0 (0)	2 190 (92)	100 (5)	37 (2)	26 (1)

F= female; M= male

**Table II:** Changes in adults’ movements in active files from 2018 to 2023

Year (Headcount)	Patients’ type				Outcome					
New n (%)	Old n (%)	Incoming transfer n (%)	Missing data n (%)	Alive n (%)	Deceased (%)	Lost to follow-up	Dropout n (%)	Outgoing transfer n (%)	Treatment discontinuation n (%)
2018 (1 780)	168 (9)	1 067 (60)	19 (1)	526 (30)	1 727 (97)	21 (1)	24 (1)	0 (0)	8 (0)	0 (0)
2019 (1 668)	184 (11)	859 (52)	17 (1)	608 (36)	1 636 (98)	14 (1)	3 (0)	0 (0)	15 (1)	0 (0)
2020 (1 883)	204 (11)	1 647 (88)	15 (1)	18 (1)	1 867 (99)	6 (0)	3 (0)	0 (0)	7 (0)	0 (0)
2021 (1 999)	115 (6)	1 868 (94)	10 (1)	6 (0)	1 887 (94)	20 (1)	69 (3)	3 (0)	19 (1)	1 (0)
2022 (2 068)	177 (9)	1 842 (89)	26 (1)	223 (1)	2 010 (99)	17 (1)	23 (1)	0	18 (1)	0
2023 (2 383)	296 (12)	2019 (86)	27 (1)	18 (1)	2 255 (95)	37 (2)	52 (2)	1 (0)	38 (2)	0

The proportion of adults with a dispensing rate of at least 95% was 61.4%, 63.6%, 70.3%, 65.9%, 57.3%, and 46.8%, respectively, from 2018 to 2023. The median treatment duration (in years) and interquartile range for the same period were 5 [2-8], 5 [2-9], 5 [2-9], 6 [3-10], 7 [3-12], and 6 [3-12], respectively. From 2018 to 2023, first-line treatment was used in 1,701 people (95.6%), 996 people (60.2%), 1,845 people (98.7%), 1,965 people (98.8%), 2,023 people (98.6%), and 2,324 people (98.8%), respectively. Treatment combinations evolved over the five-year period. Table III shows the evolution of treatment combinations used from 2018 to 2023.

**Table III:** Evolution of therapeutic combinations used in adults from 2018-2023 in the Plateau-Central region, Burkina Faso, across the active file

Year	Protocol 1	Protocol 2	Protocol 3	Protocol 4
2018 (1 780)	TDF/FTC/EFV1 (41,9 %)	AZT/3TC/NVP3 (30,8 %)	AZT/3TC/EFV6 (9,7 %)	ABC/3TC/EFV8 (0,1 %)
2019 (1 668)	TDF/FTC/EFV (49,6 %)	AZT/3TC/NVP (25,3 %)	TDF/FTC/NVP (4,6 %)	AZT/3TC/EFV (4 %)
2020 (1 883)	TDF/FTC/EFV (38,2 %)	TDF/3TC/DTG (33,1 %)	TDF/3TC/EFV4 (17,4 %)	TDF/FTC/EFV (3,7 %)
2021 (1 999)	TDF/3TC/EFV (45,7 %)	TDF/3TC/DTG (44,7 %)	TDF/FTC/EFV (3,5 %)	TDF/FTC+LPV/r (2,3 %)
2022 (2 068)	TDF/3TC/DTG (76,2 %)	TDF/3TC/EFV (23,6 %)	ABC/3TC/DTG7 (0,2 %)	ABC/3TC/EFV (0,7 %)
2023 (2 383)	TDF/3TC/DTG (90,6 %)	TDF/3TC/EFV (8,9 %)	ABC/3TC/DTG (0,1 %)	ABC/3TC/EFV (0,1 %)

TDF/FTC/EFV: ART combining Tenofovir plus Emtricitabine plus Efavirenz

TDF/3TC/DTG: ART combining Tenofovir plus Lamivudine plus Dolutegravir

AZT/3TC/NVP: ART combining Zodovudine plus Lamivudine plus Nevirapine

TDF/3TC/EFV: ART combining Tenofovir plus Lamivudine plus Efavirenz

AZT/3TC/EFV: ART combining Zidovudine plus Lamivudine plus Efavirenz

ABC/3TC/DTG: ART combining Abacavir plus Lamivudine plus Dolutegravir

ABC/3TC/EFV: ART combining Abacavir plus Lamivudine plus Efavirenz

TDF/FTC+LPV/r: ART Tenofovir plus Emtricitabine plus Lopinavir /Ritonavir

TDF/FTC/NVP : ART combining Tenofovir plus Emtricitabine plus Nevirapin

Data on RAVI6M and RACODESS were only available in the database in 2023. RAVI6M concerned 705 people (29.5%), and RACODESS concerned 231 people (10%).

The number of children in the active files monitored from 2018 to 2023 was 76, 80, 86, 92, 104, and 127, respectively. The characteristics of these children are presented in Table IV.

**Table IV:** Changes in the characteristics of children living with HIV from 2018 to 2023

Year (Headcount)	Median age of active patient file (years) [IQR]	Gender			HIV type			
F n (%)	M n (%)	Missing data n (%)	HIV1 n (%)	HIV2 n (%)	HIV1 & 2	Missing data n (%)
2018 (76)	9 [5-11]	37 (49)	34 (45)	5 (1)	69 (95)	4 (6)	0 (0)	3
2019 (80)	9 [5-11]	34 (43)	41 (51)	5 (6)	73 (97)	2 (3)	0 (0)	5
2020 (86)	8 [5-11]	40(47)	45 (52)	1 (1)	82 (97)	3 (4)	0 (0)	1
2021 (92)	8 [4-11]	38(41)	52 (57)	2 (2)	88 (97)	3 (3)	0 (0)	1
2022 (104)	8 [4-12]	42(40)	53 (51)	9 (9)	100 (98)	2 (2)	0 (0)	2
2023 (127)	10 [5-14]	57 (45)	57 (45)	10 (8)	121 (99)	1 (1)	0 (0)	5

F= female; M= male; IQR: interquartile range

Table V shows the movements of children in the active patient file.

**Table V:** Changes in children's movements in active files from 2018 to 2023

Year (Headcount)	Patients’ type				Outcome					
New n (%)	Old n (%)	Incoming transfer n (%)	Missing data n (%)	Alive n (%)	Deceased (%)	Lost to follow-up	Dropout n (%)	Outgoing transfer n (%)	Treatment discontinuation n (%)
2018 (76)	8 (11)	64 (89)	0 (0)	4 (5)	75 (99)	1 (1)	0 (0)	0 (0)	0 (0)	0 (0)
2019 (80)	12 (16)	62 (82)	2 (3)	4 (5)	79 (99)	1 (1)	0 (0)	0 (0)	0 (0)	0 (0)
2020 (86)	14 (18)	65 (81)	1 (1)	6 (7)	85 (99)	1 (1)	0 (0)	0 (0)	0 (0)	0 (0)
2021 (92)	8 (9)	84 (92)	0 (0)	0 (0)	88 (96)	3 (3)	0 (0)	0 (0)	1 (1)	0 (0)
2022 (104)	22 (22)	78 (78)	0 (0)	4 (4)	97 (93)	4 (4)	1 (1)	0 (0)	2 (2)	0 (0)
2023 (127)	37 (30)	87 (70)	0 (0)	3(2)	121 (95)	3 (2)	2 (2)	0 (0)	1 (1)	0 (0)

From 2018 to 2023, the proportion of children with at least a 95% dispensation rate was 41.1%, 75.6%, 68.6%, 66.3%, 63.3%, and 24.2%, respectively. From 2018 to 2023, the first-line treatment was used by 55 (77%), 64 (85%), 74 (99%), 79 (87%), 86 (91%), and 85 (90%) individuals, respectively. The median age and interquartile range at the start of treatment were 2 [0-9], 4 [1-7], 2 [0-9], 5 [2-10], 3 [2-7], and 4 [2-9], respectively, from 2018 to 2023. The median treatment duration from 2018 to 2023 was 3 [2-6], 44 [4-6], 4 [2-6], 4 [2-6], 4 [2-7], and 4 [1-8], respectively. Treatment combinations evolved between 2013 and 2023. Table VI provides details on the evolution of treatment combinations used in children from 2018 to 2023.

**Table VI:** Evolution of therapeutic combinations used in children from 2018-2023

Year	Protocol 1	Protocol 2	Protocol 3	Protocol 4
2018 (76)	AZT/3TC/NVP (56,7 %)	ABC/3TC/EFV (21,7 %)	ABC/3TC/LPV/r (8,3 %)	ABC/3TC/LPV/r (8,3 %)
2019 (80)	AZT/3TC/NVP (58,7 %)	ABC/3TC/EFV (17,3 %)	ABC/3TC/LPV/r (17,3 %)	ABC/3TC/NVP (4 %)
2020 (86)	AZT/3TC/NVP (40,3 %)	ABC/3TC/EFV (24,4 %)	ABC/3TC/LPV/r (19,5 %)	TDF/3TC/EFV (3,7 %)
2021 (92)	AZT/3TC/NVP (40,2 %)	ABC/3TC/LPV/r (24,1 %)	ABC/3TC/EFV (18,1 %)	TDF/3TC/DTG (6,9 %
2022 (104)	ABC/3TC/DTG (51,6 %)	AZT/3TC/NVP (18,3 %)	TDF/3TC/DTG (18,3 %)	ABC/3TC/LPV/r (9,6 %)
2023 (127)	ABC/3TC/DTG (84,3 %)	TDF/3TC/DTG (9 %)	ABC/3TC + LPV/r (2,3 %)	ABC /3TC/EFV (1,1 %)

TDF/FTC/EFV: ART combining Tenofovir plus Emtricitabine plus Efavirenz

TDF/3TC/DTG: ART combining Tenofovir plus Lamivudine plus Dolutegravir

AZT/3TC/NVP: ART combining Zodovudine plus Lamivudine plus Nevirapine

TDF/3TC/EFV: ART combining Tenofovir plus Lamivudine plus Efavirenz

ABC/3TC/DTG: ART combining Abacavir plus Lamivudine plus Dolutegravir

ABC/3TC/EFV: ART combining Abacavir plus Lamivudine plus Efavirenz

ABC/3TC/LPV/r: ART combining Abacavir plus Lamivudine plus Lopinavir / Ritonavir

ABC/3TC/NVP: ART combining Abacavir plus Lamivudine plus Nevirapine

## Discussion

This study provides an overview of antiretroviral treatment among people living with HIV/AIDS (PLHIV) in Burkina Faso during the era of the WHO “test and treat” strategy, using the Plateau-Central region as a case study. This region has not been affected by the security challenges that Burkina Faso has faced for about ten years. These challenges have not impacted PLHIV care activities or supply inputs. We assessed the proportion of people on treatment, gained an understanding of the various treatment combinations used, and observed the level of drug dispensing at the pharmacy level.

This study has methodological limitations. The data are based on dispensing records, which may contain errors related to data entry, such as incorrect dates of birth or initiation of ART. Additionally, since we did not work with the primary data sources, other errors are likely present. Despite the existence of a variable related to viral load, no data on this variable is available. Additionally, strikes in the sector significantly impacted the data in 2019.

The second UNAIDS 95 target is that 95% of people diagnosed with HIV should be on ART. Testing and treatment have helped improve results related to this target. Results show a gradual increase in the proportion of people on ART, rising from 25.84% in 2018 to over 100% in 2023. In fact, the number of people on treatment in 2023 exceeds the number of people tested. This is because not all testing data is reported to the regional level for compilation. Additionally, some people who were tested outside of the Plateau-Central region come to the region to enroll in treatment for reasons of confidentiality or proximity. Initially, the implementation of “test and treat” met resistance, particularly from doctors who opposed nurses initiating ART at care sites without conducting minimum biological tests, especially those assessing creatinine levels and the risk of kidney complications. Additionally, resistance was often noted among PLHIV due to fear of stigmatization and adverse events induced by ART, which were often reported by other PLHIV. The proportion of patients receiving treatment was inconsistent in 2021, at 40.63%. This is believed to be related to a temporary shortage of data collected in the Zorgho health district.

The median age at treatment initiation was 32 to 35 years for adults and 2 to 5 years for children. Essomba *et al.* also reported this age range among adults in a 2012 study of 1,661 PLHIV in Cameroon [3]. The age at which children were enrolled is slightly below that reported by Ngwej *et al.* in a 2015 study of a cohort of 72 children in Congo [8]. If the mode of transmission was vertical, this age at enrollment is late. Screening of children is uncommon in our context. They are often diagnosed with HIV when they develop an opportunistic infection. Activities that raise awareness about early screening should be promoted to provide optimal care for children. Furthermore, given the positive results of programs that prevent mother-to-child transmission of HIV through the use of highly active antiretroviral therapies, we should encourage more pregnant women to participate in these programs to reduce or eliminate mother-to-child transmission of HIV.

Throughout the period under review, females accounted for the majority of cases among adults. However, this trend fluctuated among children. This predominance of females is consistent with the national trend, in which HIV prevalence is higher among women than men [9]. It has also been reported in other African countries: Guinea, the Democratic Republic of the Congo, and Cameroon [1, 3, 24]. Furthermore, women living with HIV have been found to be more likely than men to have access to HIV testing and treatment [11, 12].

During the five-year study period, a number of adults were lost to follow-up, with an upward trend. Among children, the number of people lost to follow-up is low. In the context of monitoring PLHIV on ART, people lost to follow-up have always been a challenge in Burkina Faso [2, 22, 23]. ART must be taken daily for life, requiring follow-up visits and refills that may necessitate multiple trips to health facilities. This concept of active case management can be burdensome for some PLHIV, who subsequently drop out without providing any updates.

HIV-related lethality rates range from 1% to 2% among adults and from 1% to 4% among children. For adults these figures are similar to national and global data [5, 12]. However, the figures for children are higher. This slight increase could be explained by the fact that some children in our study begin ART late, particularly if their infection is not vertical. Although deaths are still recorded among PLHIV, they are significantly lower than the lethality rates reported in some studies a decade ago [17, 21, 22], demonstrating the effectiveness of ART and the quality of medical follow-up for patients. These low rates illustrate the safety of ART and their crucial role in reducing HIV-related mortality, confirming their effectiveness in combating the disease. The dispensing rate of at least 95% of ART remains modest among adults, ranging from above 50% between 2019 and 2022 to around 50% in 2022 and 2023. A similar trend is seen among children, with dispensing rates above 60% between 2019 and 2022 and below 50% in 2023. These results suggest challenges in maintaining optimal adherence over time. Although new adherence support strategies, such as RAVI6M and RACODESS, were introduced in 2022 to improve adherence among PLHIV, further efforts are needed to increase dispensing rates. Additionally, regular evaluations of these strategies’ impact are needed to better tailor them to the needs of PLHIV.

Over a six-year period, our study showed that first-line treatment was predominantly used in adults and children, reflecting good tolerance of ART without the need for second- or third-line treatment [14].

The 2013 WHO recommendations state that first-line ART should combine two nucleoside reverse transcriptase inhibitors with a non-nucleoside reverse transcriptase inhibitor for patients infected with HIV-1 [20]. Our study's results are fully in line with these recommendations, confirming their relevance in our context.

Treatment protocol evolution between 2018 and 2023 reflects a transition to more effective, better-tolerated molecules, in line with international recommendations [23]. In adults, a significant shift towards TDF/3TC/DTG occurred in 2020, reaching 90.55% by 2023.

In children, ABC/3TC + DTG became dominant in 2023, reaching 84.27%. These positive developments indicate the successful adoption of the new guidelines. However, continued analysis of resistance profiles and adherence data could optimize protocols further.

## Conclusion

This study highlights significant advances in the fight against HIV/AIDS but also reveals major challenges regarding guideline implementation, access to care equity, and optimal adherence maintenance. Particular attention must be paid to pediatric populations and adherence strategies to ensure a lasting impact in the region.

## Acknowledgments

We would like to thank Dr. Issa Ouedraogo, Secretary General of the Ministry of Health, for granting us permission to collect data. We would also like to thank the district chief medical officers, pharmacists, and state pharmacy technicians for their collaboration.

## Funding

This work did not receive any funding.

## Authors’ contributions

Dr. Zoungrana-Yameogo designed this study, analyzed and interpreted the data, and wrote the initial draft, which Dr. Christian Yonli reviewed. The text was then shared with Drs. Arielle Rita Belem, Fidèle Bakiono, Luc Delma, Mireille Ouo Coulibaly, and Abdoulaye So for review. Toussaint Compaoré participated in processing and cleaning the database. Prof. Maxime Koiné Drabo supervised this research and authorized the publication of the manuscript.

## Conflicts of interest

The authors declare no conflicts of interest.
